# Supplementary material for: Acceptability and feasibility of strategies to shield the vulnerable during the COVID-19 outbreak: a qualitative study in six Sudanese communities
Source: BMC Public Health. 2021 Jun 16;21:1153. doi: 10.1186/s12889-021-11187-9 (PMC8206886; doi:10.1186/s12889-021-11187-9)
Supplement: Supplementary file 1 — Additional file 1. Interview Guide. [file 12889_2021_11187_MOESM1_ESM.docx]

# Supplementary file 1: Interview Guide

# Acceptability and feasibility of strategies to shield the vulnerable during the COVID-19 outbreak: a qualitative study in six Sudanese communities

Nada Abdelmagid^1, 7^, Salma A.E. Ahmed^2,7^, Nazik Nurelhuda^3,7^, Israa Zainalabdeen^4,7^, Aljaile Ahmed^4,7^, Mahmoud Ali Fadlallah^5,6,7,^ Maysoon Dahab^1,7^

^1^ London School of Hygiene and Tropical Medicine, (Department of Infectious Disease Epidemiology), London, (London), United Kingdom

^2^ Independent public health researcher, Khartoum, (Khartoum), Sudan

^3^ University of Khartoum, Faculty of Dentistry, Khartoum, (Khartoum), Sudan

^4^ Y-PEER Sudan, Khartoum, (Khartoum), Sudan

^5^ Asian Institute of Technology, Bangkok, (Bangkok), Thailand

^6^ Public Health Institute (PHI), Khartoum, (Khartoum), Sudan

^7^ Sudan COVID-19 Research Group

### Corresponding author:

Nada Abdelmagid, [nada.abdelmagid@lshtm.ac.uk](mailto:nada.abdelmagid@lshtm.ac.uk)

*Notes for the interviewer: After obtaining informed consent (as set out in the standard operating procedures) and the participant is ready to begin the interview remind him or her of the following:*

- - *I will take approximately 30-40 minutes with you today*
  - *You are free to stop the interview at any time and you do not have to give me a reason*
  - *Ask them to find a quiet spot in the house, if possible, so that you can hear them clearly*
  - *I will now ask you some questions about your household and also how you and your family can protect yourself from getting coronavirus.*
  - *If you have any questions about anything, I will be happy to answer them as best as I can at the end of the interview*

1. **Household information** *(Interviewer script: I will now ask you a few questions to learn more about you, your household and your family members)*
   1. How old are you?
   2. Are you the head of household?
   3. Do you normally work outside the home? This is even if you are not going to work now because of the stay-at-home orders.
   4. If yes, what work do you do outside the home?
   5. How many people live in your household?
   6. Does any of your household members go out regularly (once per week or more) for work or school or shopping?
   7. How many people in your household fit into the following categories:
      1. 50 years or older OR
      2. Regardless of age, has one of the following conditions: a chronic disease (diabetes, hypertension, kidney failure, cancer, heart disease or respiratory disease) or an illness that reduces that immunity.
2. **COVID-19 knowledge** *(Interviewer script: Now I would like to ask you some questions about a disease called coronavirus)*
   1. When did you first hear about coronavirus?
   2. What have you heard about it?
   3. How worried are you that you or someone in your family could get coronavirus? Why?
3. **Knowledge about high risk groups** *(Interviewer script: now I would like to discuss with you people who are 50 years or older or who have an underlying medical condition (e.g. diabetes, hypertension, heart or respiratory illness) regardless of how they are. Let us call them the high-risk individuals)*
   - 1. Have you heard that high risk people might be more likely to develop serious illness if they get coronavirus? If yes, what have you heard and where did you hear that from?
4. **Acceptability and feasibility of shielding** *(Interviewer script: I’d like to ask you now your opinion about how to protect people who are high risk from coronavirus. But first let me tell you some the advice from medical experts for those individuals. Medical experts say the following:*
   - *Those who are 50 years of age or older or those with a chronic illness or who are immunocompromised (regardless of age) have a higher risk of serious illness if they get coronavirus.*
   - *The medical advice is that people and especially those at high risk (older and with pre-existing conditions) stay home as much as possible and reduce or eliminate their interaction with others outside their house.*
   - *They also advise that if anyone who has to go out regularly for shopping and work and lives with a high-risk person must wash their hands before entering the home so they don’t bring coronavirus infection in the house. Also, they need to distance themselves and not prepare food or drinks for high-risk people just in case they’re asymptomatic.*
   1. Have you heard about this advice before? If yes, where did you hear it from?
   - *Others public health experts have said that in countries like Sudan where appropriate medical care is not widely available that it is important to be extra careful about protecting those who are at high risk from coronavirus infection.*
   - *They suggest that those at high risk should be isolated from other members of their household if any of their household members continue to go out regularly for shopping and work.*
   1. What are your thoughts on ways to protect those who are at higher risk of serious illness from getting coronavirus infection?
   2. *Interviewer script: I will now tell you some options that have been suggested to protect high risk people. I would like to know what you think about each option, specifically whether you think your community can carry out these measures given the space and resources they have.*
      1. Firstly, what do you think about the suggestion each household can choose one person to go out regularly for work and shopping and that that person be isolated from the rest of the family when they are at home to protect high risk family members.

***(Pause for response)***

- - 1. Secondly, what do you think about the suggestion that each household create a separate space for high risk people and anyone who cares for them to sleep and eat separately from the rest of the family

***(Pause for response)***

- - 1. Thirdly, what do you think about the suggestion that, where there is not enough space in the household to separate the high risk from the low risk individuals, multiple families (either neighbours or extended families) swap houses to give high risk individuals and any who has to care for them a separate house to live in without having to go out for food or work and others who are at low risk can stay in a different house and continue to work as necessary

***(Pause for response)***

- - 1. In such cases those at low risk and living in other households will have to deliver food and other necessities so high-risk individuals living together do not have to leave home for food or work.
    2. Do you have any other suggestion for other ways to protect those who are at higher risk serious illness from getting coronavirus infection? If yes, what are those ideas?

1. **Communication channels** *(Interviewer script: We are trying to understand, from talking to people like you, how we can inform people across Sudan about coronavirus and especially about how they can protect high risk individuals. Now I would like to ask your opinion on how we can deliver such public health messages):*
   1. What do you think are the best way to explain to people across Sudan that there are some people who are at high risk from serious illness from coronavirus and that they need to be given extra protection from infection?
   2. Who would you trust to deliver this information to you *(interviewer prompt: is it someone in your community, family, or health officials?)*
   3. Can you rank these people you mentioned, from most to least influential?
   4. What communication channels should we use the most in the community *(interviewer prompt: e.g. through other community members, tv, radio, social media. If they say all, ask them to rank them in terms of importance).*
