# Supplementary material for: Acceptability and feasibility of strategies to shield the vulnerable during the COVID-19 outbreak: a qualitative study in six Sudanese communities
Source: BMC Public Health. 2021 Jun 16;21:1153. doi: 10.1186/s12889-021-11187-9 (PMC8206886; doi:10.1186/s12889-021-11187-9)
Supplement: Supplementary file 2 — Additional file 2. Training materials for data collection. [file 12889_2021_11187_MOESM2_ESM.docx]

# Supplementary file 1: Training materials for data collection

# Acceptability and feasibility of strategies to shield the vulnerable during the COVID-19 outbreak: a qualitative study in six Sudanese communities

Nada Abdelmagid^1, 7^, Salma A.E. Ahmed^2,7^, Nazik Nurelhuda^3,7^, Israa Zainalabdeen^4,7^, Aljaile Ahmed^4,7^, Mahmoud Ali Fadlallah^5,6,7,^ Maysoon Dahab^1,7^

^1^ London School of Hygiene and Tropical Medicine, (Department of Infectious Disease Epidemiology), London, (London), United Kingdom

^2^ Independent public health researcher, Khartoum, (Khartoum), Sudan

^3^ University of Khartoum, Faculty of Dentistry, Khartoum, (Khartoum), Sudan

^4^ Y-PEER Sudan, Khartoum, (Khartoum), Sudan

^5^ Asian Institute of Technology, Bangkok, (Bangkok), Thailand

^6^ Public Health Institute (PHI), Khartoum, (Khartoum), Sudan

^7^ Sudan COVID-19 Research Group

### Corresponding author:

Nada Abdelmagid, [nada.abdelmagid@lshtm.ac.uk](mailto:nada.abdelmagid@lshtm.ac.uk)

Introduction to Qualitative Research: <https://youtu.be/xdR7xAk_Dyg>

Introduction to COVID-19 and Shielding: <https://youtu.be/uSHU46y2trQ>

Informed Consent: <https://youtu.be/pi0cIsiQNqc>

Study Objectives and Procedures: <https://youtu.be/2YhX0C4jAsE>
